# Serum glutamine, set-shifting ability and anorexia nervosa

**DOI:** 10.1186/1744-859X-9-29

**Published:** 2010-06-25

**Authors:** Michiko Nakazato, Kenji Hashimoto, Ulrike Schmidt, Kate Tchanturia, Iain C Campbell, David A Collier, Masaomi Iyo, Janet Treasure

**Affiliations:** 1 Section of Eating Disorders, Institute of Psychiatry, King's College London, UK; 2 Department of Child Psychiatry, Chiba University Hospital, Chiba, Japan; 3 Division of Clinical Neuroscience, Chiba University Center for Forensic Mental Health, Chiba, Japan; 4 Division of Psychological Medicine and Social Genetic and Developmental Psychiatry Centre, Institute of Psychiatry, King's College London, UK; 5 Department of Psychiatry, Chiba University Graduate School of Medicine, Chiba, Japan; 6 Division of Psychological Medicine, Eating Disorders Research Unit, Department of Academic Psychiatry, King's College, Guy's Hospital, London, UK

## Abstract

**Background:**

Set-shifting is impaired in people with anorexia nervosa (AN), but the underlying physiological and biochemical processes are unclear. Animal studies have established that glutamatergic pathways in the prefrontal cortex play an important role in set-shifting ability. However, it is not yet understood whether levels of serum glutamatergic amino acids are associated with set-shifting performance in humans. The aim of this study was to determine whether serum concentrations of amino acids related to glutamatergic neurotransmission (glutamine, glutamate, glycine, l-serine, d-serine) are associated with set-shifting ability in people with acute AN and those after recovery.

**Methods:**

Serum concentrations of glutamatergic amino acids were measured in 27 women with current AN (AN group), 18 women recovered from AN (ANRec group) and 28 age-matched healthy controls (HC group). Set-shifting was measured using the Wisconsin Card Sorting Test (WCST) and the Trail Making Task (TMT). Dimensional measures of psychopathology were used, including the Eating Disorder Examination Questionnaire (EDEQ), the Maudsley Obsessive-Compulsive Inventory (MOCI) and the Hospital Anxiety and Depression Scale (HADS).

**Results:**

Serum glutamine concentrations in the AN group (1,310.2 ± 265.6 μM, mean ± SD) were significantly higher (by approximately 20%) than those in the HC group (1,102.9 ± 152.7 μM, mean ± SD) (*F*_(2, 70) _= 6.3, *P *= 0.003, 95% CI 61.2 to 353.4). Concentrations of serum glutamine were positively associated with markers of the illness severity: a negative correlation was present between serum glutamine concentrations and body mass index (BMI) and lowest BMI and a positive correlation was found between duration of illness and EDEQ. The AN group showed significantly impaired set shifting in the WCST, both total errors, and perseverative errors. In the AN group, there were no correlations between serum glutamine concentrations and set shifting.

**Conclusions:**

Serum concentrations of glutamine may be a biomarker of illness severity in people with AN. It does not appear to be directly associated with changes in executive function.

## Background

Specific cognitive characteristics have been observed in people with eating disorders (ED) [1,2]. For example, set-shifting difficulties have been found in people currently ill with anorexia nervosa (AN), in an attenuated form in people recovered from AN (ANRec) [3,4] and in unaffected sisters [5]. The problem has also been identified in bulimia nervosa (BN), schizophrenia [6], bipolar disorder [7] and obsessive-compulsive disorder [8]. It appears to be a trait as it is present in first-degree relatives of people with schizophrenia [9] and bipolar disorder [10].

Glutamate is the principal excitatory neurotransmitter in brain and is involved in cognitive functions such as memory and learning [11]. As glutamate concentrations in blood are correlated with those in cerebrospinal fluid (CSF) [12,13], serum levels may influence glutamatergic concentrations and functions in brain. This is of interest because muscle breakdown and gluconeogenesis during starvation is likely to increase serum glutamine. This proposal has some indirect support from proton magnetic resonance spectroscopy (MRS) studies, which have reported that people with AN have lower levels of a combined measure of glutamate and glutamine (Glx) and of *N*-acetyl aspartate (NAA) in the frontal grey matter [14]. Furthermore, executive functioning assessed using the Wisconsin Card Sorting Test (WCST) has been shown to be associated with Glx levels in the anterior cingulate gyrus (ACC) [15]. It has also been proposed that the age-related decline in set-shifting ability is associated with alterations in glutamate receptor binding in the cingulate cortex and dorsomedial striatum [16]. These various studies suggest that the functioning of the glutamatergic system in the prefrontal region may be related to the impaired cognitive performance seen in people with AN and in other psychiatric disorders [17,18]. Animal studies support the idea that set shifting is associated with glutamatergic neurotransmission (for example, with *N*-methyl-d-aspartate (NMDA) receptor function) [16,19-21]. MRS studies of people with AN [14,15,22,23] have shown heterogenous findings possibly due to methodological factors.

Based on the above findings, we hypothesised that, firstly, alterations in serum concentrations of glutamatergic amino acids (glutamate, glutamine, glycine, l-serine and d-serine) would be observed in individuals with AN and those with recovered AN and, secondly, that such alterations would be related to deficits in set-shifting ability in individuals with acute AN and those with recovered AN.

## Methods

### Participants

Of the 73 women who participated in this study, 27 had current AN (AN group), 18 had recovered from AN (ANRec group) and 28 were healthy age-matched controls (HC group) (Table 1). Individuals in the AN and ANRec groups were recruited from the South London and Maudsley National Health Service (NHS) Foundation Trust volunteer register of individuals with past or current ED. The HC group was recruited from volunteers in the local community.

**Table 1:** Clinical characteristics and findings of serum amino acids (one-way ANOVA)

	AN (n = 27)	ANRec (n = 18)	HC (n = 28)	*F*	df	*P *value
Age, years	27.7 ± 10.6	32.2 ± 11.1	26.9 ± 5.8	2.0	2, 70	0.14
Education, years	16.9 ± 3.2	17.0 ± 2.8	18.1 ± 2.1	1.5	2, 66	0.23
Duration, years	10.0 ± 10.6	6.6 ± 6.2	NA	13.7	2, 69	0.00
Age of onset, years	17.0 ± 4.4	18.4 ± 6.4	NA	152.7	2, 69	0.00
Current BMI, kg/m^2^	15.4 ± 1.6^a^**^b^**	19.8 ± 1.1^b^**^c^**	22.3 ± 2.5	85.6	2, 68	0.00
Lowest BMI, kg/m^2^	13.1 ± 1.6^a^**	14.2 ± 2.0^c^**	21.0 ± 2.4	111.2	2, 65	0.00
EDEQ R	4.3 ± 3.7^a^**^b^**	0.8 ± 0.9^b^**	0.6 ± 1.0	20.0	2, 68	0.00
EDEQ E	3.3 ± 1.5^a^**^b^**	0.4 ± 0.4^b^**	0.2 ± 0.2	86.9	2, 68	0.00
EDEQ W	4.1 ± 1.7^a^**^b^**	1.0 ± 0.9^b^**	0.5 ± 0.6	69.8	2, 68	0.00
EDEQ S	4.6 ± 1.6^a^**^b^**	1.5 ± 0.9^b^**	0.9 ± 0.7	80.2	2, 68	0.00
EDEQ G	3.9 ± 1.7^a^**^b^**	1.0 ± 0.8^b^**	0.5 ± 0.5	65.7	2, 67	0.00
MOCI	10.1 ± 6.0^a^**	8.7 ± 4.2^c^**	3.7 ± 2.8	14.6	2, 68	0.00
HADS anxiety	14.0 ± 4.4^a^**^b^**	8.7 ± 2.8^b^**^c^**	4.4 ± 3.3	45.9	2, 66	0.00
HADS depression	9.3 ± 5.5^a^**^b^**	3.4 ± 2.4^b^**	1.5 ± 2.6	28.7	2, 66	0.00
Serum Glu, μM	64.7 ± 32.3	45.0 ± 29.6	54.7 ± 23.1	2.7	2, 70	0.08
Serum Gln, μM	1,310.2 ± 265.6^a^**	1,159.0 ± 236.3	1,102.9 ± 152.7	6.3	2, 70	0.00
Serum glycine, M	294.8 ± 78.2	280.9 ± 82.9	255.1 ± 58.3	2.1	2, 70	0.13
Ratio of Glu/Gln	0.064 ± 0.065	0.042 ± 0.034	0.052 ± 0.025	1.3	2, 70	0.28
Serum d-serine, μM	2.2 ± 0.8	2.2 ± 0.8	1.9 ± 0.5	1.6	2, 70	0.21
Serum l-serine, μM	135.6 ± 53.7	158.4 ± 72.7	117.7 ± 60.9	2.4	2, 70	0.10

Values shown are mean ± SD; *P < 0.05; **P < 0.01.

^a^Comparisons between AN and HC; ^b^comparisons between AN and ANRec; ^c^comparisons between ANRec and HC.

AN = anorexia nervosa; ANOVA = analysis of variance; ANRec = recovered from anorexia nervosa; BMI = body mass index; df = degrees of freedom; EDEQ R = Eating Disorder Examination Questionnaire Restraint Subscale; EDEQ E = Eating Concern Subscale; EDEQ W = Weight Concern Subscale; EDEQ S = Shape Concern Subscale; EDEQ G = Global Scale; Glu = Glutamate; Gln = Glutamine; HADS = Hospital Anxiety and Depression Scale; HC = healthy controls; MOCI = Maudsley Obsessive-Compulsive Inventory.

All participants in the AN group met the American Psychological Society (APA) *Diagnostic and Statistical Manual of Mental Disorders, fourth edition *(DSM-IV) criteria [24] for AN (20 with the restrictive subtype, 7 with the binge-purge subtype). Seven patients were diagnosed with major depressive disorders; one also had an anxiety disorder and two had concurrent obsessive compulsive disorders. The ANRec group was defined according to the following criteria: (1) a history of AN of the restrictive subtype as defined by DSM-IV, (2) maintenance of a stable body mass index (BMI) between 18.5 and 24 kg/m^2 ^for a minimum of 1 year, (3) regular menstrual cycles (at least 10 cycles) during the past year, (4) binge eating and purging behaviours absent for 1 year, and (5) not having been prescribed any psychotropic medication during the past year. Inclusion criteria for the HC group were: (1) BMI between 19 and 26 kg/m^2^, (2) no personal or family history of any psychiatric illness or ED, and (3) no current use of psychotropic medication. Groups were matched for age, ethnicity and educational level.

Exclusion criteria for all participants included a history of brain injury, psychosis, neurological or other severe medical illness, alcoholism or drug abuse/dependence. All participants had English as their first language. Ethical approval for the study was obtained from the Institute of Psychiatry and the South London and Maudsley NHS Trust Research Ethics Committee. All participants provided written informed consent for participation in the study.

### Clinical and self-report measures of psychopathology

Current and lowest previous BMI was recorded for patients in the AN and ANRec groups. Neuropsychological assessments were carried out in all but three AN patients. Dimensional measures of psychopathology were used, including the Eating Disorder Examination Questionnaire (EDEQ) [25], which has four subscales of Restraint (EDEQ-R), Eating Concern (EDEQ-E), Weight Concern (EDEQ-W) and Shape Concern (EDEQ-S). The Maudsley Obsessive Compulsive Inventory (MOCI) [26] and the Hospital Anxiety and Depression Scale (HADS) [27] were used as dimensional measures to assess current anxiety, depression and obsessive-compulsive symptoms.

### Assay of serum glutamatergic amino acids

Blood samples were drawn from all subjects by venepuncture in the morning (9:00 to 12:00). Approximately 10 ml of peripheral venous blood was collected into additive-free containers and the samples were stored at -80°C until needed.

Measurement of amino acids was carried out using methods described previously [18,28,29]. Serum levels of glutamate, glutamine, and glycine were measured using high performance liquid chromatography (HPLC) [28]. d-Serine and l-serine levels were determined by a column switching HPLC system with fluorescence detection [30]. A total of 20 μl of the human serum was homogenised in 180 μl of HPLC-grade methanol. Homogenates were then centrifuged at 4,500 *g *for 10 min. Then, 20 μl of supernatant was evaporated to dryness at 40°C and the residue was rehydrated by adding 20 μl of H_2_O (HPLC grade), 20 μl of 0.1 M borate buffer (pH 8.0) and 60 μl of 50 mM 4-fluoro-7-nitro-2,1,3-benzoxadiazole (NBD-F; Tokyo Kasei Kogyo, Tokyo, Japan) in CH_3_CN (HPLC grade). The reaction mixture was then heated at 60°C for 1 min, and immediately supplemented with 100 μl of H_2_O/CH_3_CN (90/10) containing 0.1% trifluoroacetic acid (TFA) to stop the reaction. A total of 10 μl of the resultant solution was injected into the HPLC system [28,29].

### Assessment of set-shifting ability

The WCST [31] and the Trail Marking Task (TMT) [32] were used to assess executive function by the measurement of set-shifting ability.

The WCST involves matching stimulus cards with one of four category cards. The sorting rule (colour, shape or number) changes unpredictably after 10 correct sorts. The set-shifting outcome employed is the number of raw perseverative errors.

The TMT is a traditional set-shifting task. It requires participants to connect an alphabetical sequence on a page in a 'dot-to-dot' fashion (trail A), before alternatively linking numbers and letters in order (that is, 1-A-2-B-3-C (trail B)). A computerised version of the TMT was employed here [33]. The set-shifting outcome used was a balanced variable of trail B minus trail A, to control for baseline motor speed.

### Statistical analysis

All data were analysed using SPSS V.17.0 for Windows (SPSS, Chicago, IL, USA). Results are presented as mean values ± standard deviation (SD). Two-way analysis of variance (ANOVA) was carried out to test for the interaction between the groups of participants, cognitive impairment of set-shifting abilities and serological findings. One-way ANOVA was used to test for differences in clinical characteristics, neuropsychological tasks and serum amino acids concentrations between the groups. Where a significant overall difference between the groups was observed in ANOVA, pairwise comparisons were carried out using the Bonferroni-Dunn *post hoc *test to test the significance of different combinations of groups with respect to the outcome variables. Pearson's bivariate correlation coefficients were calculated to examine the relationship between serum concentrations of the different glutamatergic amino acids with clinical variables (age, education, duration of illness, current BMI, lowest BMI), and also with results from the neuropsychiatric dimensional tests (EDEQ subscale, MOCI and HADS anxiety and depression scores). The values of Cohen's *d *were calculated to be 0.20, 0.50 and 0.80 (small, medium and large effect size, respectively), and *P *values < 0.05 were considered statistically significant.

## Results

### Demographic and clinical characteristics

Table 1 shows the demographic and clinical characteristics for all participants. There were no significant differences between the AN group and ANRec group in terms of current age, years of education, age of illness onset, duration of illness or lowest BMI. The ANRec group had been recovered for a mean duration of 7.2 years (SD 6.4; range 1 to 24). As expected, the AN group had a significantly lower BMI and a significantly higher level of psychopathology (as determined by the EDEQ and HADS anxiety and depression scores) than the ANRec and the HC groups. The effect sizes were calculated to be 1.16 for EDEQ-R, 1.56 for HADS anxiety and 1.38 for HADS depression scores. The ANRec group showed significantly higher levels of anxiety on MOCI and HADS anxiety testing compared with the HC group.

### Serum concentrations of amino acids

Two-way ANOVA revealed no between-subjects effects of group or set shifting on serum concentration of amino acids.

**Figure 1:**
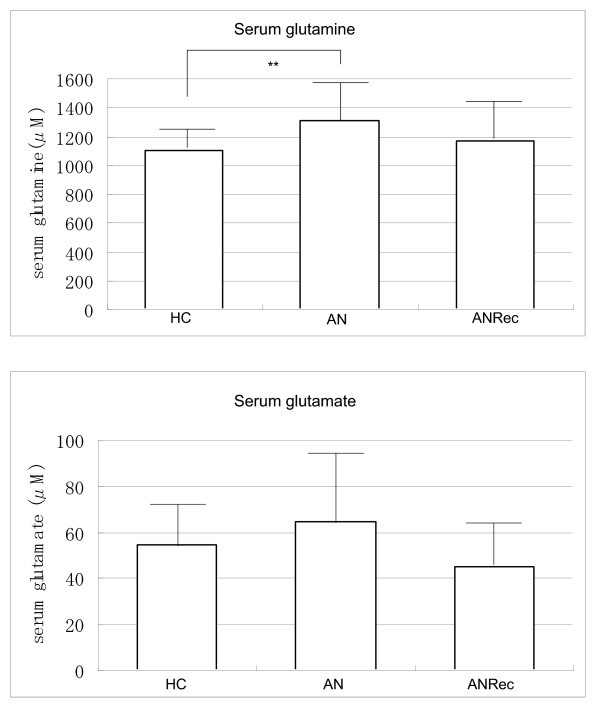
**Comparison of serum glutamine concentrations and serum glutamate concentrations in the healthy controls (HC), the patients with anorexia nervosa (AN) and those recovered from AN (ANRec)**. Values are mean ± SD; **P < 0.01.

Table 1 shows the concentrations of amino acids between the three groups. Serum glutamine concentrations in the AN group (n = 27) (1,310.2 ± 265.6 μM, mean ± SD) were significantly higher than those in the HC group (n = 28) (1,102.9 ± 152.7 μM, mean ± SD) (*F*_(2, 70) _= 6.3; *P *= 0.003) (Figure 1). Table 2 shows the results of the *post hoc *Bonferroni-Dunn test for serum glutamine concentrations. Serum glutamine concentrations were significantly higher in the AN group than in the HC group (*P *= 0.003; 95% CI 61.2 to 353.4). The effect size for the mean differences in serum glutamine was 0.87, which is a large effect. There were no significant differences in the serum concentrations of the other amino acids (glutamate, glycine, d-serine and l-serine) between the three groups. The effect sizes for these were of a small and medium size: 0.36 for glutamate, 0.56 for glycine, 0.44 for d-serine and 0.31 for l-serine, respectively.

**Table 2:** *Post hoc *Bonferroni tests for the serum glutamine concentrations

Group		95% CI	*P *value
AN (n = 27)	HC (n = 28)	61.2 to 353.4	0.003*
	ANRec (n = 18)	-13.6 to 316	0.083
HC (n = 28)	ANRec (n = 18)	-219.7 to 107.5	1.000

One-way ANOVA, *Post hoc *Bonferroni test.

*P < 0.05.

AN = anorexia nervosa; ANOVA = analysis of variance; ANRec = recovered from anorexia nervosa; HC = healthy controls.

### Neuropsychological findings

Group comparisons for the neuropsychological tasks are presented in Table 3. The AN group showed significantly impaired set shifting on the WCST (both total errors and perseverative errors). The effect sizes were 0.71 for the total errors and 0.68 for perseverative errors. The scores for the ANRec group were between those of the AN group and the HC group, and this difference was not statistically significant.

**Table 3:** Neuropsychological findings of set shifting (performance on the WCST and the Trail Making Task (TMT))

	AN (n = 24)	ANRec (n = 18)	HC (n = 28)
Trail shifting time	33.3 ± 17.6	29.6 ± 11.3	29.1 ± 8.6
Trail shifting errors	2.0 ± 6.5	0.6 ± 1.0	0.4 ± 0.6
WCST total errors, %	24.0 ± 15.6^a^**	17.6 ± 8.5	14.9 ± 7.7
WCST perseverative errors, %	13.0 ± 10.4^a^**	8.7 ± 3.5	7.6 ± 3.4

Values are mean ± SD; *P < 0.05; **P < 0.001.

^a^Comparisons between AN and HC; ^b^comparisons between AN and ANRec; ^c^comparisons between ANRec and HC.

AN = anorexia nervosa; ANRec = recovered from anorexia nervosa; HC = healthy controls; WCST = the Wisconsin Card Sorting Test.

### Correlations between serum glutamine concentrations, cognitive function and psychopathological features of EDs

In the sample that included the AN group and the ANRec group (n = 45), a negative correlation was found between serum glutamine concentrations and BMI (*P *= 0.026; *r *= -0.339), and lowest BMI (*P *= 0.01; *r *= -0.386). A positive correlation was found between serum glutamine concentrations and HADS anxiety scores (*P *= 0.005; *r *= 0.433). There were also positive correlations between serum glutamate concentrations and EDEQ-W scores (*P *= 0.006; *r *= 0.413) and EDEQ-S (*P *= 0.03; *r *= 0.332). In the AN group (n = 27), there was a positive correlation between serum glutamate and the scores on EDEQ-W (*P *= 0.048; *r *= 0.399).

### Regression analysis

To investigate the relative importance of measured variables as predictors of eating-related psychopathology, multiple regression analyses of selected variables (serum d-serine, serum l-serine, serum glycine, serum glutamine, serum glutamate and glutamate/glutamine ratio) were carried out on WCST, EDEQ, HADS anxiety and HADS depression scores. Stepwise regression analysis indicated that serum glutamate levels in the AN group predicted EDEQ-W scores. In the total sample, stepwise regression analyses also indicated that serum glutamine levels predicted anxiety and depression. When glutamine was eliminated from this regression model, 18.8% of the variance that predicted anxiety scores was explained (R^2 ^= 0.188, *P *= 0.012, 95% CI -13.1 to 2.7, β = 0.434) along with 21.4% of the variance that predicted depression scores (R^2 ^= 0.214, β = 0.462, *P *= 0.007). In the AN group, one component, serum glutamate was extracted and found to explain 90.8% of the predictive variance of EDEQ-W scores (R^2 ^= 0.908, *P *= 0.047, 95% CI -2.14 to 4.431, β = 0.953). No other variables were found to predict ED clinical components or set-shifting performance.

## Discussion

In this study, we found that serum glutamine concentrations in women currently ill with AN were significantly higher than in a healthy control group of women. The effect size for the mean differences in serum glutamine was 0.87, which is a large effect size. The effect sizes were 0.36 for serum glutamate, 0.56 for serum glycine, 0.44 for d-serine, 0.31 for l-serine: these are between a small and a medium size. Secondly, our data shows that elevated concentrations of serum glutamine are associated with illness severity. For example, serum glutamine concentrations were negatively correlated with BMI and lowest BMI, and there was a positive correlation between the serum glutamine concentration and duration of illness, and also the EDEQ score. As elevated serum glutamine concentrations are likely to be derived from muscle breakdown and gluconeogenesis during starvation, it is suggested that increased serum glutamine is a state marker for the physiological severity of the AN. Our second hypothesis, namely that serum glutamine concentrations would be related to impairment of set-shifting abilities in people with AN was not confirmed.

Depression has a lifetime prevalence of 5% to 10% of young women and has a high comorbidity with AN. Previous studies found that plasma levels of glutamine, glutamate were significantly increased in female patients with depression [33,34]. Given that depression may reflect disturbances in glutamatergic activity, screening HC controls on psychiatric history might bias the results and that the screening for exclusion should have been based on history of ED only.

In this study, there were no significant differences in the levels of the glutamine/glutamate ratio between the AN and the HC group (Table 1). The amino acid glutamine is involved in glutamate uptake, and although this study was not designed as a turnover study, we hypothesised that we would be able to recognise an altered glutamatergic cycle in patients with AN. The levels of serum glutamine in the AN group were found to be higher than those in the HC group. One possibility is that in severe AN, raised serum glutamine is a compensatory metabolic response for having decreased levels in the brain due to malnutrition.

The main endogenous source of circulating glutamine is de novo synthesis in striated muscle via the enzyme glutamine synthetase (GS). In animal studies, GS plays a key role in mounting the adaptive response to fasting by transiently facilitating the production of glutamine [35]. Intracellular concentrations of amino acids in the skeletal muscle of healthy non-obese people decrease markedly during fasting; after 3 days of fasting the glutamine concentrations are seen to have fallen [36]. The previous report showed that in AN, reduced body protein could be confirmed by measurement of the triceps skinfold thickness [37]. Taken together, elevated serum glutamine appears to be derived from muscle breakdown and gluconeogenesis during starvation, which in turn is related to BMI and duration of illness. Our second hypothesis that serum glutamatergic amino acids would be related to cognitive impairment of set-shifting abilities in people with AN was not confirmed.

In this study, the AN group showed significantly impaired set-shifting in the WCST, both total errors and perseverative errors. The scores in the recovered group were inbetween those of participants in the acute phase of the illness and HC. Neuropsychological function using WCST was worse in AN participants in comparison with the control group, which was similar to the findings of previous studies [1-5,38].

The limitations of this study were a small sample size and a cross-sectional design. Thus we could not conclude whether serum glutamatergic neurotransmission were associated with set-shifting difficulties both in acute AN and ANRec. A longitudinal study is required, using a larger sample size and exploring other central coherence tasks, in order to clarify whether glutamatergic amino acids are a biological markers for certain endophenotypes of AN.

Finally, it is unclear whether serum glutamatergic concentrations in humans accurately reflect levels in the brain. Such concentrations might represent breakdown of muscle in the periphery, as products of gluconeogenesis, rather than reflect changing levels in the brain. Further studies are required to confirm what alterations in glutamatergic neurotransmission occur in the brain of individuals with AN, and how it relates to the pathophysiology. This could be performed using MRS to directly assess the levels of glutamine in the frontal grey matter.

## Conclusions

Elevated serum glutamine may be related to the pathophysiology of AN but does not appear to be linked to functional changes in executive function. Further longitudinal studies are required to explore the associations between glutamatergic amino acid metabolism and cognitive flexibility in AN.

## Competing interests

The authors declare that they have no competing interests.

## Authors' contributions

MN wrote the protocol and carried out the recruitment of the participants, performed the statistical analyses. KH participated in the design and carried out assay of glutamatergic amino acids. US participated in the design, coordination of the study. KT participated in coordination of the neuropsychological assessment. ICC participated in the design, the interpretation of data, revised the manuscript draft. DAC participated in the design, the management of blood samples. MI participated in the design of the study. JT participated in its design and coordination of the study. All authors participated in the interpretation of data, revised it critically for important intellectual content and have read and approved the final manuscript.
